# The Expression Patterns of FAM83H and PANX2 Are Associated With Shorter Survival of Clear Cell Renal Cell Carcinoma Patients

**DOI:** 10.3389/fonc.2019.00014

**Published:** 2019-01-22

**Authors:** Kyoung Min Kim, Usama Khamis Hussein, Jun Sang Bae, See-Hyoung Park, Keun Sang Kwon, Sang Hoon Ha, Ho Sung Park, Ho Lee, Myoung Ja Chung, Woo Sung Moon, Myoung Jae Kang, Kyu Yun Jang

**Affiliations:** ^1^Department of Pathology, Chonbuk National University Medical School, Jeonju, South Korea; ^2^Research Institute of Clinical Medicine of Chonbuk National University, Jeonju, South Korea; ^3^Biomedical Research Institute of Chonbuk National University Hospital, Jeonju, South Korea; ^4^Research Institute for Endocrine Sciences, Chonbuk National University, Jeonju, South Korea; ^5^Faculty of Science, Beni-Suef University, Beni-Suef, Egypt; ^6^Department of Bio and Chemical Engineering, Hongik University, Sejong, South Korea; ^7^Department of Preventive Medicine, Chonbuk National University Medical School, Jeonju, South Korea; ^8^Division of Biotechnology, Chonbuk National University, Iksan, South Korea; ^9^Department of Forensic Medicine, Chonbuk National University Medical School, Jeonju, South Korea

**Keywords:** kidney, clear cell renal cell carcinoma, FAM83H, PANX2, prognosis

## Abstract

FAM83H is primarily known for its role in amelogenesis; however, recent reports suggest FAM83H might be involved in tumorigenesis. Although the studies of FAM83H in kidney cancer are limited, a search of the public database shows a significant association between FAM83H and pannexin-2 (PANX2) in clear cell renal cell carcinomas (CCRCCs). Therefore, we evaluated the clinicopathological significance of the immunohistochemical expression of FAM83H and PANX2 in 199 CCRCC patients. The expression of FAM83H and PANX2 were significantly associated with each other. In univariate analysis, individual, and co-expression pattern of FAM83H and PANX2 was significantly associated with shorter overall survival (OS) and relapse-free survival (RFS) of CCRCC patients: nuclear expression of FAM83H (OS; *P* < 0.001, RFS; *P* < 0.001), cytoplasmic expression of FAM83H (OS; *P* < 0.001, RFS; *P* < 0.001), nuclear expression of PANX2 (OS; *P* < 0.001, RFS; *P* < 0.001), cytoplasmic expression of PANX2 (OS; *P* < 0.001, RFS; *P* < 0.001), co-expression pattern of nuclear FAM83H and nuclear PANX2 (OS; *P* < 0.001, RFS; *P* < 0.001). In multivariate analysis, nuclear expression of FAM83H (OS; *P* < 0.001, RFS; *P* = 0.003) and the co-expression pattern of nuclear FAM83H and PANX2 (OS; *P* < 0.001, RFS; *P* < 0.001) were independent indicators of shorter survival of CCRCC patients. Cytoplasmic expression of FAM83H was associated with shorter RFS (*P* = 0.030) in multivariate analysis. In Caki-1 and Caki-2 CCRCC cells, knock-down of FAM83H decreased PANX2 expression and cell proliferation, and overexpression of FAM83H increased PANX2 expression and cell proliferation. These results suggest that FAM83H and PANX2 might be involved in the progression of CCRCC in a co-operative manner, and their expression might be used as novel prognostic indicators for CCRCC patients.

## Introduction

Family with sequence similarity 83 H (FAM83H) is one of the eight FAM83 family members, from FAM83A to FAM83H (1, 2). According to the type of FAM83, diverse roles for members of FAM83 family have been reported (1, 2). Among them, FAM83H is characterized by its essential roles in dental enamel formation, and mutation of FAM83H was causative in autosomal dominant hypocalcified amelogenesis imperfecta (3–5). Recently, in addition to the role of FAM83H in amelogenesis, its role in tumorigenesis has been reported (1, 6, 7). Increased gene expression of FAM83H has been reported in various human cancers such as pulmonary adenocarcinomas, pulmonary squamous cell carcinomas, breast cancers, hepatocellular carcinomas, ovarian cancers, pancreatic cancers, and gastric cancers (1). In prostate cancer cells, the expression of FAM83H was associated with the proliferation of cancer cells and increased risk of recurrence of prostate cancer patients (8). In colon cancer cells, FAM83H was involved in regulation of keratin organization (7, 9). In addition, the expression of FAM83H was transcriptionally controlled by the oncogene MYC and associated with proliferation and invasiveness of liver cancer cells (6). Furthermore, the expression of FAM83H was an independent indicator of poor prognosis of hepatocellular carcinoma patients (6) and higher expression of FAM83H was associated with shorter disease-free survival of uterine cancer (1). In addition, among the eight FAM83 family members, the expression of FAM83D and FAM83H was consistently higher in tumors in various human tissue types, with up-regulation of FAM83D observed in 78% of tumors (21 of 27 data sets) and up-regulation of FAM83H observed in 59% of tumors (16 of 27 data sets) (1). However, controversially, gene expression of FAM83H was decreased in some brain tumors and higher expression of FAM83H was associated with the favorable prognosis of glioblastoma and head and neck cancers (1). Since the role of FAM83H in tumorigenesis might differ according to the type of tissue or cancer, further study is needed to clarify its role.

Kidney cancer is common in highly-developed countries, and clear cell renal cell carcinoma (CCRCC) is the most common type of kidney cancer (10). Most CCRCCs are sporadic, and some genetic alterations have been identified (10). Recent studies have focused on finding novel biomarkers and several systemic treatment modalities targeting cell proliferation, tumor angiogenesis, and tumor cell survival have been developed (10). However, further study is needed to identify specific molecular targets involved in the development and progression of CCRCCs. However, despite increasing interest in the role of FAM83H in human cancers, to date, no study has focused on kidney cancer. Therefore, we investigated the possible role of FAM83H in CCRCC, the most common type of kidney cancer. The possibility that FAM83H is involved in the progression of CCRCC was indicated by a search of the OncoLnc public database (http://www.oncolnc.org). In addition, the cBioPortal database (http://www.cbioportal.org) indicated pannexin-2 (PANX2) as the gene most significantly associated with FAM83H expression in CCRCCs (11, 12). Although it has been reported that the expression of PANX2 was very low in kidney tissue, the expression of PANX2 mRNA was the gene most significantly associated with the expression of FAM83H mRNA in CCRCC (cBioPortal database; http://www.cbioportal.org, Pearson's correlation; 0.71, Spearman's correlation; 0.36, accession date; 26 August 2018) (11, 12). PANX2 is a member of the pannexin family, which consists of Panx1, PANX2, and Panx3. The study of PANX2 has been limited to its function in the central nervous system because PANX2 is primarily expressed herein (13). However, in a recent analysis of the distribution of PANX2 in various type of tissue, PANX2 was expressed ubiquitously in mouse tissue, including kidney, where it was observed in the renal tubular epithelium (14). Therefore, based on the higher correlation between the expression of FAM83H and PANX2 in public databases, we evaluated the expression and prognostic significance of FAM83H and PANX2 expression in 199 CCRCCs.

## Materials and Methods

### Patients and Samples

This study evaluated CCRCC patients who underwent surgical resection between July 1998 and August 2011. Among them, 199 CCRCCs with complete medical records were available with original H&E slides, and paraffin-embedded tissue blocks. Clinical information was obtained by reviewing the medical records. The histopathologic information was retrospectively reviewed according to the 2016 World Health Organization classification of the renal tumors (15), and staged according to the staging system of the American Joint Committee on Cancer (16). The clinicopathologic factors evaluated in this study were sex, age, tumor size, tumor stage, lymph node metastasis, Fuhrman nuclear grade (17), and microscopic tumor necrosis. Median follow-up duration was 57 months (range; 1–168 months). This study obtained institutional review board approval from Chonbuk National University Hospital (IRB No., CUH 2017-09-017) and was performed according to the Declaration of Helsinki. The approval contained a waiver for written informed consent based on the retrospective and anonymous character of this study.

### Immunohistochemical Staining and Scoring

In this study, tissue microarrays were used for immunohistochemical staining. The tissue microarrays have 3.0 mm cores, and one core was arrayed per case from the areas composed mainly of tumor cells with no degenerative change or necrosis. The histologic sections from the tissue microarray tissue blocks were deparaffinized and boiled for 20 min in pH 6.0 antigen retrieval solution (DAKO, Glostrup, Denmark) in a microwave oven. Primary antibodies for FAM83H (1:100, Bethyl Laboratories, Montgomery, TX) and PANX2 (1:100, Novus Biologicals, Littleton, CO) was used. The slides were visualized with the enzyme substrate 3-amino-9-ethylcarbazole and counterstained with hematoxylin. The scoring for the immunohistochemical staining slides was performed without clinical information by two pathologists (KYJ and KMK) with consensus under a multi-viewing microscope. The immunohistochemical positivity for FAM83H and PANX2 was separately scored according to their expression patterns in the cytoplasm and nuclei. The immunohistochemical scores were obtained by adding their staining intensity score ranging from zero to three (0; no staining, 1; weak, 2; intermediate, 3; strong) and staining area score ranging from zero to five (0; no staining, 1; 1%, 2; 2–10%, 3: 11–33%, 4; 34–66%, 5; 67–100%) (6, 18, 19). Therefore, the immunohistochemical staining scores ranged from zero to eight.

### Clear Cell Renal Cell Carcinoma Cells and Cell Culture

In this study, we used two human CCRCC cell lines. Caki-1 and Caki-2 cells were purchased from the Korean Cell Line Bank (KCLB, Seoul, Korea). The Caki-1 and Caki-2 cells were cultured in DMEM medium with 10% fetal bovine serum (Gibco BRL, Gaithersburg, MD) and streptomycin and penicillin (100 U/ml) in 5% CO_2_ and 37°C.

### Plasmids and Transfection

The vector of shRNA for FAM83H was purchased from GenePharma (Shanghai, China). The sense and antisense sequences of the FAM83H shRNA were the 5′-CAC CGC TCA TCT TCA GCA CGT CAC ATT CAA GAG ATG TGA CGT GCT GAA GAT GAG CTT TTT TG-3′ and 5′-GAT CCA AAA AAG CTC ATC TTC AGC ACG TCA CAT CTC TTG AAT GTG ACG TGC TGA AGA TGA GC-3′, respectively. The vector for FAM83H overexpression (Catalog #, EX-Y4473-M03; accession #, NM_198488) was purchased from GeneCopoeia (Rockville, MD). JetPRIME transfection reagent (Polyplus Transfection, Illkirch, France) was used for transfection.

### Western Blot Analysis

To isolate protein, the cells were lysed with PRO-PREP Protein Extraction Solution (iNtRON Biotechnology Inc., Korea) with 1x phosphatase inhibitor cocktails 2, 3 (Sigma-Aldrich). The primary antibodies for FAM83H (Bethyl Laboratories, Montgomery, TX), PANX2 (1:100, Novus Biologicals, Littleton, CO), and actin (Sigma-Aldrich) were used for western blots.

### Quantitative Reverse-Transcription Polymerase Chain Reaction

RNA was isolated with an RNeasy Mini Kit (Qiagen Sciences, Valencia, CA). The isolated RNA was reverse transcribed by probing 1.5 μg RNA with Reverse Transcription kits (Applied Biosystems, Foster City, CA) to generate cDNA that was used for quantitative polymerase chain reaction using Applied Biosystems Prism 7900HT sequence Detection System and SYBR Green Master PCR Mix (Applied Biosystems). The values were normalized to the expression of the glyceraldehyde-3-phosphate dehydrogenase reference housekeeping gene. All experiments were performed in triplicate. The sequences of the primers used in quantitative reverse-transcription polymerase chain reaction are listed in Table 1.

**Table 1:** Primer sequences used for quantitative real-time polymerase chain reaction.

**Gene**	**Primer sequence forward/reverse**	**Product size**	**Accession number**
*FAM83H*	F: 5′-CATGGTCCAGACAACCTGTG-3′	214	NM_198488.3
	R: 5′-GCTGGATACCAGGAGGACAA-3′		
*PANX2*	F: 5′-AGAAGGCCAAGACTGAGGCG-3′	124	NM_001160300.1
	R: 5′-GGAGCATCTTTGGTGGGTGC-3′		
*CCND1* (Cyclin D1)	F: 5′-GAGGAAGAGGAGGAGGAGGA-3′	236	NM_053056.2
	R: 5′-GAGATGGAAGGGGGAAAGAG-3′		
*CCNE1* (Cyclin E1)	F: 5′-AGCGGTAAGAAGCAGAGCAG-3′	189	NM_001238.3
	R: 5′-TTTGATGCCATCCACAGAAA-3′		
*CDKN1B* (p27)	F: 5′-AGATGTCAAACGTGCGAGTG-3′	154	NM_004064.4
	R: 5′-TCTCTGCAGTGCTTCTCCAA-3′		
*GAPDH*	F: 5′-AACAGCGACACCCACTCCTC-3′	258	NM_001256799.1
	R: 5′-GGAGGGGAGATTCAGTGTGGT-3′		

*Web link to accession numbers: https://www.ncbi.nlm.nih.gov/gene*.

### Cell Proliferation Assay

The proliferation of cells was evaluated by counting the number of cells and performing a 3-(4,5-dimethylthiazol-2-yl)-2,5-diphenyltetrazonium bromide (MTT) (Sigma-Aldrich, St. Louis, MO) assay and a colony-forming assay. To compare the number of cells in control, FAM83H knock-down, and FAM83H overexpression groups, Caki-1 (2 × 10^5^) and Caki-2 (2 × 10^5^) cells were seeded in 24-well plates and the number of viable cells was counted by using hemocytometer. For the MTT assay, Caki-1 (3 × 10^3^) and Caki-2 (3 × 10^3^) cells were seeded in 96-well culture plates, and absorbance was measured at 560 nm using a microtiter plate reader (Bio-Rad, Richmond, CA).

### Statistical Analysis

The cut-off points of immunohistochemical staining scores for FAM83H and PANX2 were determined by receiver operating characteristic curve analysis (6, 19). Survival analysis was performed for overall survival (OS) and relapse-free survival (RFS), and the follow-up endpoint was December 2012. OS duration was calculated as the time from the date of diagnosis to the date of death or last contact. The patients who died from CCRCC were treated as events for OS analysis. The patients who died from other causes, lacked follow-up, or who were alive at last contact were treated as censored for OS analysis. RFS duration was calculated as the time from the date of diagnosis to the date of relapse, death, or last contact. The patients who experienced relapse or death from CCRCC were treated as events for RFS analysis. The patients who died from other causes, lacked follow-up, or who were alive without relapse at last contact were treated as censored for RFS analysis. Survival analysis was performed by univariate and multivariate Cox proportional hazards regression analyses. To further illustrate the impact of OS and RFS, Kaplan-Meier survival analysis was performed and presented in survival curves. The associations between clinicopathologic factors and the expression of FAM83H and PANX2 were analyzed by Pearson's chi-square test and the student's *t*-test. SPSS software (IBM, version 20.0, CA) was used throughout, and *P* < 0.05 were considered statistically significant.

## Results

### The Expression Patterns of FAM83H and PANX2 Are Associated With Advanced Clinicopathological Characteristics of CCRCCs

**Figure 1:**
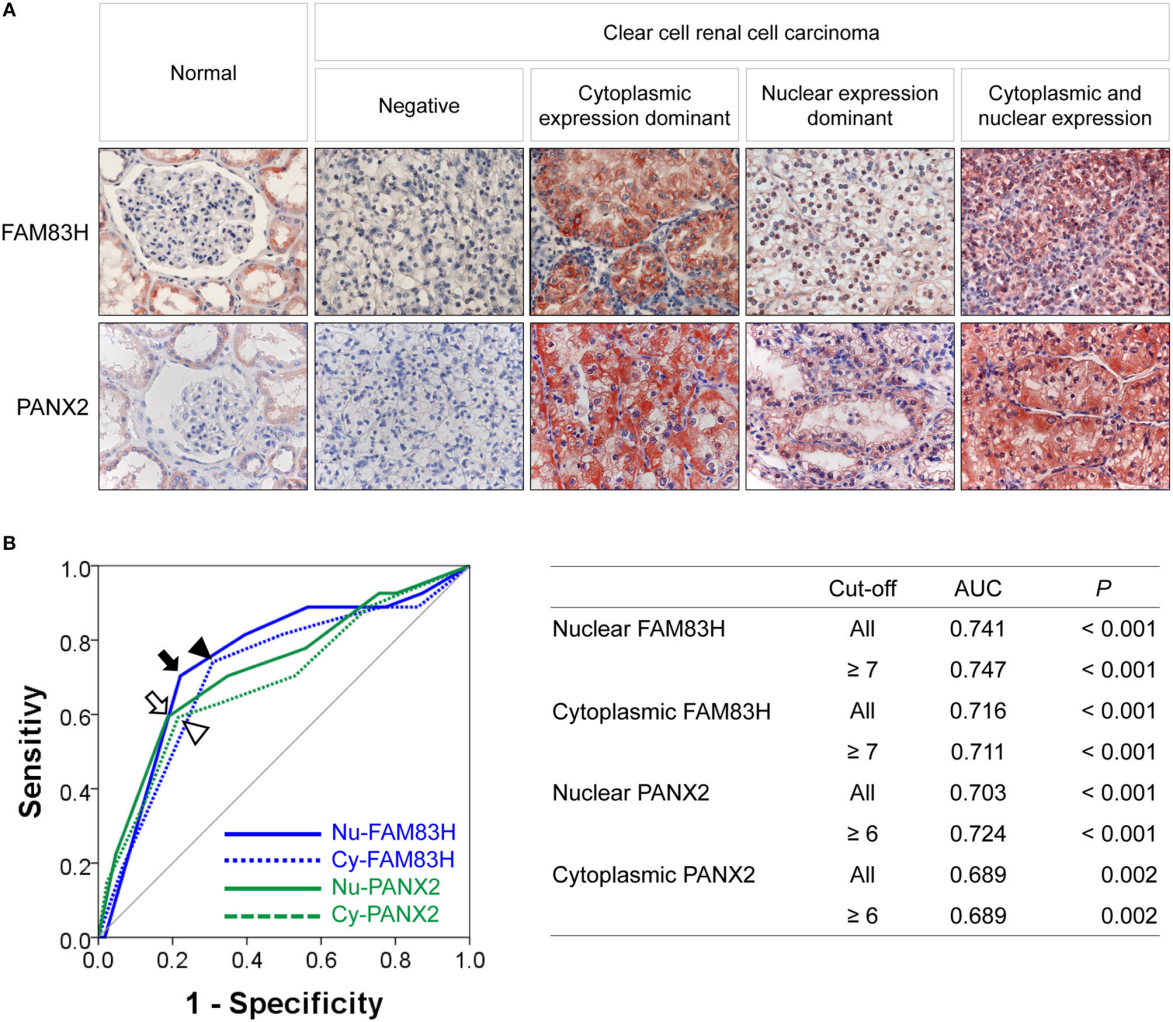
Immunohistochemical expression of FAM83H and PANX2 in clear cell renal cell carcinomas and statistical analysis. **(A)** Immunohistochemical expression of FAM83H and PANX2 in normal renal parenchyma and clear cell renal cell carcinoma cells. In normal tissue, FAM83H and PANX2 are expressed in the cytoplasm of renal tubular epithelium. In clear cell renal cell carcinoma cells, FAM83H and PANX are expressed both in the cytoplasm and the nuclei. Original magnification; x400. **(B)** The cut-off values for nuclear FAM83H (Nu-FAM83H), cytoplasmic FAM83H (Cy-FAM83H), nuclear PANX2 (Nu-PANX2), and cytoplasmic PANX2 (Cy-PANX2) expression for classifying as negative- and positive-subgroups were determined by receiver operating characteristic curve analysis. The cut-off points were determined at the point with the highest area under the curve (AUC) to estimate death of clear cell renal cell carcinoma patients. The arrow (Nu-FAM83H), arrowhead (Cy-FAM83H), empty arrow (Nu-PANX2), and empty arrowhead (Cy-PANX2) indicate the cut-off points on the receiver operating characteristic curve. The cut-off point for Nu-FAM83H and Cy-FAM83H was seven, and the cut-off point for Nu-PANX2 and Cy-PANX2 was six.

FAM83H and PANX2 were detected in non-neoplastic renal tubules but not in glomerular cells (Figure 1A). In CCRCCs, immunohistochemical expression of FAM83H and PANX2 was seen in both the cytoplasm and nuclei of tumor cells (Figure 1A). In this study, we separately evaluated the cytoplasmic and nuclear expression of FAM83H and PANX2. The cut-off points determined by receiver operating characteristic curve analysis were seven for both the nuclear expression of FAM83H (Nu-FAM83H) and the cytoplasmic expression of FAM83H (Cy-FAM83H) (Figure 1B). The cut-off points for both the nuclear expression of PANX2 (Nu-PANX2) and the cytoplasmic expression of PANX2 (Cy-PANX2) were six (Figure 1B). With these cut-off values, Nu-FAM83H (*P* < 0.001), Cy-FAM83H (*P* < 0.001), Nu-PANX2 (*P* < 0.001), and Cy-PANX2 (*P* = 0.002) was significantly associated with death of patients from CCRCC (Figure 1B). Nu-FAM83H positivity was significantly associated with larger tumor size (*P* < 0.001) and higher tumor stage (*P* = 0.001) (Table 2). Cy-FAM83H positivity was significantly associated with older age of patients (*P* = 0.004), larger tumor size (*P* < 0.001), higher tumor stage (*P* < 0.001), and higher histologic grade (*P* = 0.037) (Table 2). Nu-PANX2 was significantly associated with sex (*P* = 0.009), tumor size (*P* = 0.001), tumor stage (*P* < 0.001), and tumor necrosis (*P* = 0.003) (Table 2). Cy-PANX2 was significantly associated with age of patients (*P* = 0.032), tumor size (*P* < 0.001), tumor stage (*P* < 0.001), lymph node metastasis (*P* = 0.018), nuclear grade (*P* = 0.006), and tumor necrosis (*P* = 0.011) (Table 2). Moreover, there were significant associations between Nu-FAM83H, Cy-FAM83H, Nu-PANX2, and Cy-PANX2 positivity (Table 2).

**Table 2:** Clinicopathologic variables and the expression of FAM83H and PANX2 in clear cell renal cell carcinomas.

**Characteristics**		**No**.	**Nu-FAM83H**		**Cy-FAM83H**		**Nu-PANX2**		**Cy-PANX2**	
			**Positive**	***P***	**Positive**	***P***	**Positive**	***P***	**Positive**	***P***
Sex	Male	140	44 (31%)	0.181	51 (36%)	0.909	41 (29%)	0.009	40 (29%)	0.341
	Female	59	13 (22%)		22 (37%)		7 (12%)		13 (22%)	
Age, y	≤ 55	81	20 (25%)	0.307	20 (25%)	0.004	16 (20%)	0.233	15 (19%)	0.032
	> 55	118	37 (31%)		53 (45%)		32 (27%)		38 (32%)	
Tumor size, cm	≤ 7	167	40 (24%)	< 0.001	52 (31%)	< 0.001	33 (20%)	0.001	33 (20%)	<0.001
	> 7	32	17 (53%)		21 (66%)		15 (47%)		20 (63%)	
TNM stage	I	161	38 (24%)	0.001	48 (30%)	< 0.001	31 (19%)	< 0.001	30 (19%)	<0.001
	II–IV	38	19 (50%)		25 (66%)		17 (45%)		23 (61%)	
LN metastasis	Absence	197	56 (28%)	0.502	71 (36%)	0.062	47 (24%)	0.390	51 (26%)	0.018
	Presence	2	1 (50%)		2 (100%)		1 (50%)		2 (100%)	
Nuclear grade	1	36	9 (25%)	0.367	16 (44%)	0.037	6 (17%)	0.064	5 (14%)	0.006
	2	120	32 (27%)		36 (30%)		26 (22%)		29 (24%)	
	3 and 4	43	16 (37%)		22 (51%)		16 (37%)		19 (44%)	
Necrosis	Absence	171	47 (27%)	0.372	60 (35%)	0.248	35 (20%)	0.003	40 (23%)	0.011
	Presence	28	10 (36%)		13 (46%)		15 (54%)		13 (46%)	
Cy-PANX2	Negative	146	33 (23%)	0.002	40 (27%)	< 0.001	14 (10%)	< 0.001		
	Positive	53	24 (45%)		33 (62%)		34 (64%)			
Nu-PANX2	Negative	151	31 (21%)	< 0.001	45 (30%)	< 0.001				
	Positive	48	26 (54%)		28 (58%)					
Cy-FAM83H	Negative	126	17 (13%)	< 0.001						
	Positive	73	40 (55%)							

*Nu-FAM83H, nuclear expression of FAM83H; Cy-FAM83H, cytoplasmic expression of FAM83H; Nu-PANX2, nuclear expression of PANX2; Cy- PANX2, cytoplasmic expression of PANX2*.

### Expression Patterns of FAM83H and PANX2 Were Associated With Shorter Survival of CCRCC Patients

In univariate Cox proportional hazards regression analysis, the factors significantly associated with OS of CCRCC patients were age (*P* = 0.006), tumor size (*P* < 0.001), tumor stage (*P* < 0.001), tumor necrosis (*P* = 0.006), Nu-FAM83H (*P* < 0.001), Cy-FAM83H (*P* < 0.001), Nu-PANX2 (*P* < 0.001), and Cy-PANX2 (*P* < 0.001) (Table 3). The factors significantly associated with RFS of CCRCC patients were sex (*P* = 0.033), age (*P* = 0.022), tumor size (*P* < 0.001), tumor stage (*P* < 0.001), lymph node metastasis (*P* = 0.010), nuclear grade (*P* = 0.006), Nu-FAM83H (*P* < 0.001), Cy-FAM83H (*P* < 0.001), Nu-PANX2 (*P* < 0.001), and Cy-PANX2 (*P* < 0.001) (Table 3).

**Table 3:** Univariate Cox proportional hazards regression analysis for the overall survival and relapse-free survival of clear cell renal cell carcinomas.

**Characteristics**	**No**.	**OS**		**RFS**	
		**HR (95% CI)**	***P***	**HR (95% CI)**	***P***
Sex, male (vs. female)	140/199	0.404 (0.140–1.169)	0.094	0.359 (0.139–0.923)	0.033
Age, > 55 (vs. ≤ 55)	118/199	4.450 (1.538–12.877)	0.006	2.421 (1.137–5.153)	0.022
Tumor size, > 7 cm (vs. ≤ 7 cm)	32/199	4.759 (2.226–10.173)	<0.001	5.750 (2.984–11.078)	<0.001
TNM stage, II-IV (vs. I)	38/199	4.926 (2.312–10.493)	<0.001	6.603 (3.417–12.758)	<0.001
LN metastasis, presence (vs. absence)	2/199	0.049 (0–1.034 × 10^6^)	0.726	15.800 (1.956–127.648)	0.010
Nuclear grade, 1	36/199	1	0.062	1	0.006
2	120/199	1.751 (0.398–7.711)	0.459	1.572 (0.463–5.337)	0.468
3 and 4	43/199	4.005 (0.876–18.303)	0.074	4.356 (1.251–15.170)	0.021
Necrosis, presence (vs. absence)	28/199	3.179 (1.389–7.276)	0.006	1.995 (0.908–4.381)	0.085
Nu-FAM83H positive (vs. negative)	57/199	6.205 (2.715–14.181)	<0.001	4.949 (2.504–9.780)	<0.001
Cy-FAM83H positive (vs. negative)	73/199	4.617 (1.950–10.933)	<0.001	5.369 (2.523–11.426)	<0.001
Nu-PANX2 positive (vs. negative)	48/199	4.283 (1.984–9.248)	<0.001	3.131 (1.627–6.026)	<0.001
Cy-PANX2 positive (vs. negative)	53/199	3.713 (1.717–8.033)	<0.001	3.432 (1.774–6.640)	<0.001
Nu-FAM83H/Nu-PANX2, −/− or −/+	142/199	1	<0.001	1	<0.001
+/−	31/199	2.840 (0.928–8.686)	0.067	2.922 (1.210–7.053)	0.017
+/+	26/199	10.779 (4.513–25.745)	<0.001	7.951 (3.754–16.844)	<0.001

**Figure 2:**
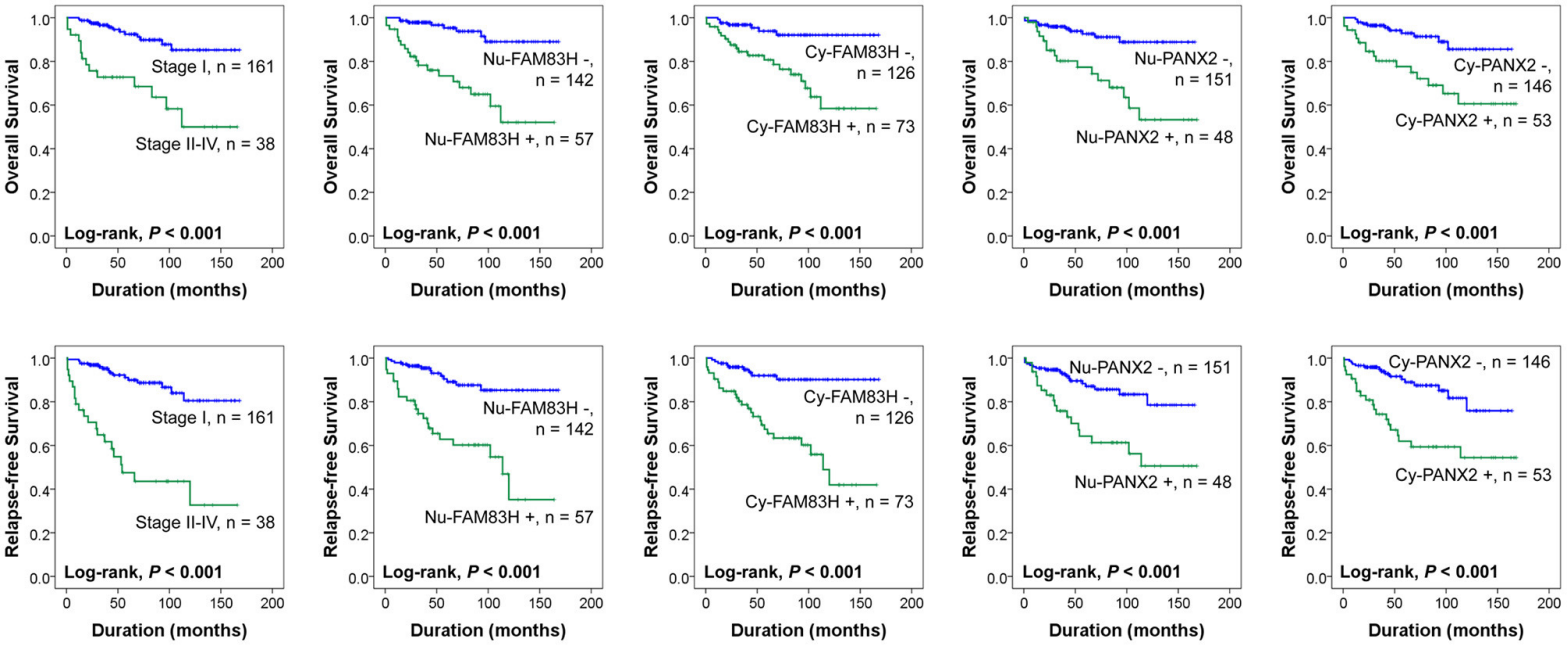
Kaplan-Meier survival analysis of 199 clear cell renal cell carcinoma patients. Kaplan-Meier survival analysis for the overall survival and relapse-free survival according to the tumor stage, nuclear expression of FAM83H (Nu-FAM83H), cytoplasmic expression of FAM83H (Cy-FAM83H), nuclear expression of PANX2 (Nu-PANX2), and cytoplasmic expression of PANX2 (Cy-PANX2).

Nuclear positivity of FAM83H expression had a 6.205-fold (95% confidence interval [95% CI]; 2.715–14.181) greater risk of death and a 4.949-fold (95% CI; 2.504–9.780) greater risk of relapse or death of CCRCC patients. Cy-FAM83H positivity showed a 4.617-fold (95% CI; 1.950–10.933) greater risk of death and a 5.369-fold (95% CI; 2.523–11.426) greater risk of relapse or death of CCRCC patients (Table 3). Nu-PANX2 positivity showed a 4.283-fold (95% CI; 1.984–9.248) greater risk of death and a 3.131-fold (95% CI; 1.627–6.026) greater risk of relapse or death of CCRCC patients. Cy-PANX2 positivity showed a 3.713-fold (95% CI; 1.717–8.033) greater risk of death and a 3.432-fold (95% CI; 1.774–6.640) greater risk of relapse or death of CCRCC patients (Table 3). The Kaplan-Meier survival curves for the OS and RFS according to tumor stage (OS; Log-rank, *P* < 0.001, RFS; Log-rank, *P* < 0.001), Nu-FAM83H (OS; Log-rank, *P* < 0.001, RFS; Log-rank, *P* < 0.001), Cy-FAM83H (OS; Log-rank, *P* < 0.001, RFS; Log-rank, *P* < 0.001), Nu-PANX2 (OS; Log-rank, *P* < 0.001, RFS; Log-rank, *P* < 0.001), and Cy-PANX2 positivity (OS; Log-rank, *P* < 0.001, RFS; Log-rank, *P* < 0.001) are presented in Figure 2.

Multivariate analysis was performed with the factors significantly associated with OS or RFS in the univariate analysis. Therefore, the factors included in the multivariate analysis were sex, age, tumor size, tumor stage, LN metastasis, histologic nuclear grade, tumor necrosis, and the expression of Nu-FAM83H, Cy-FAM83H, Nu-PANX2, and Cy-PANX2. The factors predicting OS of CCRCC patients in multivariate analysis were age (*P* = 0.047), tumor stage (*P* = 0.018), tumor necrosis (*P* = 0.031), and Nu-FAM83H positivity (*P* < 0.001) (Table 4). Tumor stage (*P* < 0.001), Nu-FAM83H positivity (*P* = 0.003), and Cy-FAM83H positivity (*P* = 0.030) were the factors significantly associated with RFS of CCRCC patients in multivariate analysis (Table 4). The patients with Nu-FAM83H-positive CCRCC had a 4.883-fold (95% CI; 2.085–11.433) greater risk of death and a 2.967-fold (95% CI; 1.457–6.045) greater risk of relapse or death. The patients with Cy-FAM83H-positive CCRCC had a 2.460-fold (95% CI; 1.091–5.547) greater risk of relapse or death (Table 4).

**Table 4:** Multivariate Cox regression analysis for the overall survival and relapse-free survival of clear cell renal cell carcinomas.

**Characteristics**	**OS**		**RFS**	
	**HR (95% CI)**	***P***	**HR (95% CI)**	***P***
Age, > 55 (vs. ≤ 55)	3.022 (1.017–8.982)	0.047		
TNM stage, II-IV (vs. I)	2.628 (1.178–5.865)	0.018	4.024 (2.019–8.016)	<0.001
Necrosis, presence (vs. absence)	2.600 (1.090–6.199)	0.031		
Nu-FAM83H positive (vs. negative)	4.883 (2.085–11.433)	<0.001	2.967 (1.457–6.045)	0.003
Cy-FAM83H positive (vs. negative)			2.460 (1.091–5.547)	0.030

*The factors significantly associated with OS or RFS were included in multivariate analysis. The variables included in the multivariate analysis was sex, age, tumor size, tumor stage, LN metastasis, histologic nuclear grade, tumor necrosis, and the expression of Nu-FAM83H, Cy-FAM83H, Nu-PANX2, and Cy-PANX2*.

### Co-expression Patterns of Nuclear FAM83H and Nuclear PANX2 Expression Predicted Shorter Survival of CCRCC Patients

**Figure 3:**
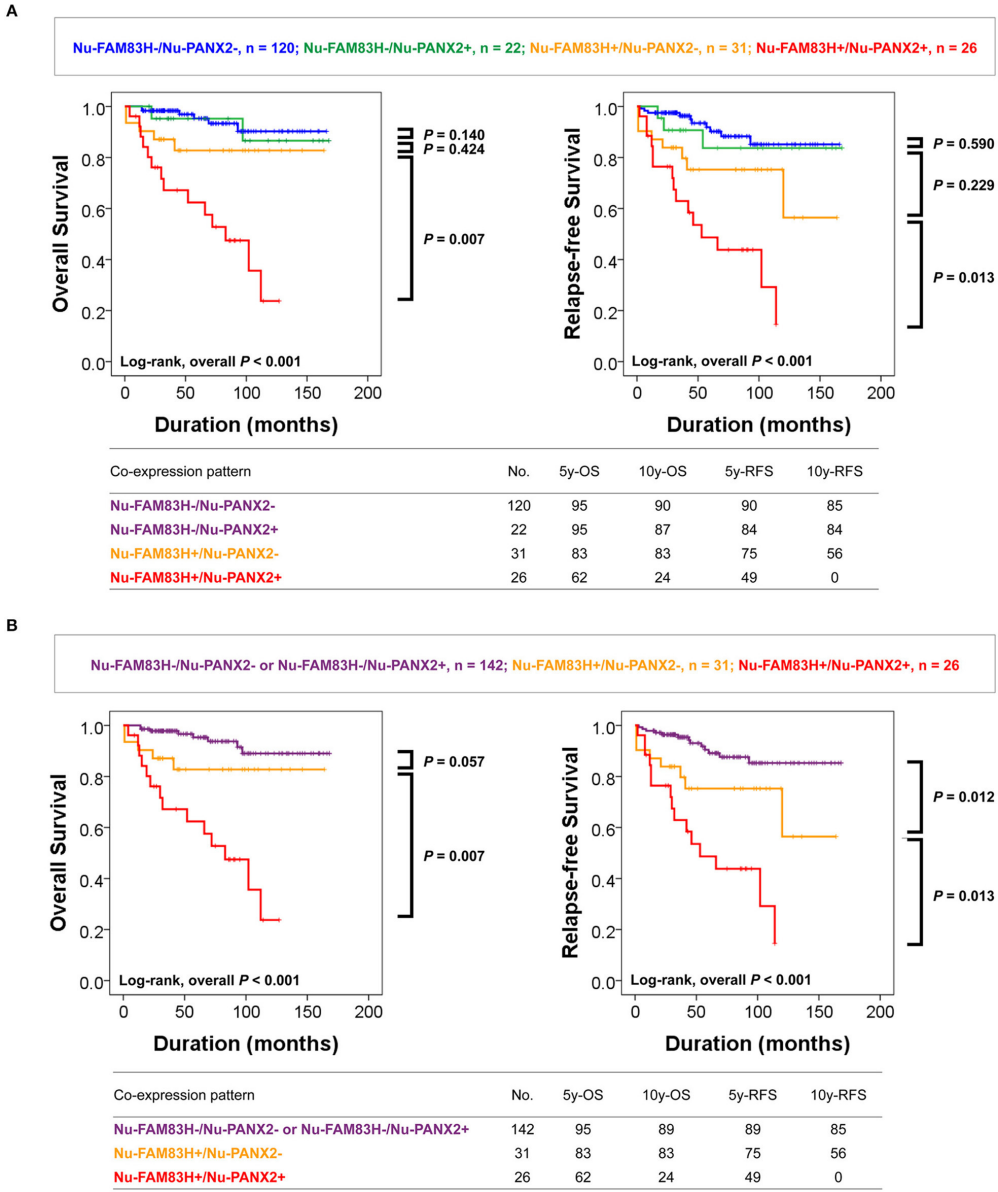
Survival analysis according to the co-expression patterns of nuclear FAM83H and nuclear PANX2 expressions. **(A)** Kaplan-Meier survival analysis in Nu-FAM83H^−^/Nu-PANX2^−^, Nu-FAM83H^−^/Nu-PANX2^+^, Nu-FAM83H^+^/Nu-PANX2^−^, and Nu-FAM83H^+^/Nu-PANX2^+^ subgroups according to the nuclear expression of FAM83H and PANX2. **(B)** Kaplan-Meier survival analysis in three subgroups according to the co-expression patterns of nuclear FAM83H and PANX2; favorable (Nu-FAM83H^−^/Nu-PANX2^−^ and Nu-FAM83H^−^/Nu-PANX2^+^), intermediate (Nu-FAM83H^+^/Nu-PANX2^−^), and poor prognostic (Nu-FAM83H^+^/Nu-PANX2^+^) subgroups. 5y-OS; five-year overall survival rate, 10y-OS; ten-year overall survival rate; 5y-RF; five-year relapse-free survival rate, 10y-RFS; ten-year relapse-free survival rate.

As we have shown in Table 2, there was a significant association between Nu-FAM83H, Cy-FAM83H, Nu-PANX2, and Cy-PANX2 positivity. In addition, Nu-FAM83H and Nu-PANX2 positivity were more predictive for estimating survival of CCRCC patients compared with Cy-FAM83H and Cy-PANX2 positivity. Therefore, we further analyzed the prognostic significance of the co-expression patterns of Nu-FAM83H and Nu-PANX2. As shown in Figure 3A, Nu-FAM83H^−^/ Nu-PANX2^−^, and Nu-FAM83H^−^/ Nu-PANX2^+^ subgroups showed a relatively favorable prognosis and the Nu-FAM83H^+^/Nu -PANX2^+^ subgroup showed a poor prognosis. The Nu-FAM83H^+^/Nu-PANX2^−^ subgroup showed intermediate survival. Based on these results, we sub-classified the co-expression patterns of Nu-FAM83H and Nu-PANX2 as favorable (Nu-FAM83H^−^/Nu-PANX2^−^ and Nu-FAM83H^−^/Nu-PANX2^+^), intermediate (Nu-FAM83H^+^/Nu-PANX2^−^), and poor prognostic (Nu-FAM83H^+^/Nu-PANX2^+^) groups that were significantly associated with OS (overall *P* < 0.001) and RFS (overall *P* < 0.001) in univariate analysis (Table 5) (Figure 3B). With this subgrouping, the 5y-OS and 10y-OS rates of Nu-FAM83H^−^/Nu-PANX2^−^ and Nu-FAM83H^−^/Nu-PANX2^+^ subgroups were 95 and 89%, respectively. The 5y-RFS and 10y-RFS rates of Nu-FAM83H^−^/Nu-PANX2^−^ and Nu-FAM83H^−^/Nu-PANX2^+^ subgroups were 89 and 85%, respectively. The Nu-FAM83H^+^/Nu-PANX2^−^ subgroup had an 83% 5y-OS rate and an 83% 10y-OS rate. The Nu-FAM83H^+^/Nu-PANX2^−^ subgroup had a 75% 5y-RFS rate and a 56% 10y-RFS rate. The 5y-OS and 10y-OS rates of the Nu-FAM83H^+^/Nu-PANX2^+^ subgroup were 62 and 24%, respectively. The 5y-RFS and 10y-RFS rates of the Nu-FAM83H^+^/Nu-PANX2^+^ subgroup were 49 and 0%, respectively (Figure 3B). When we performed multivariate analysis with inclusion of sex, age, tumor size, tumor stage, LN metastasis, histologic nuclear grade, tumor necrosis, and co-expression patterns of Nu-FAM83H and Nu-PANX2, the Nu-FAM83H/Nu-PANX2 expression patterns were independent indicators of OS and RFS (Table 5). The Nu-FAM83H^+^/Nu-PANX2^+^ subgroup had a 7.242-fold (95% CI; 2.876–18.237, *P* < 0.001) greater risk of OS and 4.903-fold (95% CI; 2.230–10.780, *P* < 0.001) greater risk of RFS compared with the Nu-FAM83H^−^/Nu-PANX2^−^ and Nu-FAM83H^−^/Nu-PANX2^+^ subgroups (Table 5). The Nu-FAM83H^+^/Nu-PANX2^−^ subgroup also showed a 180% (95% CI; 0.911–8.607, *P* = 0.072) greater risk of lower OS and a 160% (95% CI; 1.072–6.280, *P* = 0.034) greater risk of lower RFS compared with the Nu-FAM83H^−^/Nu-PANX2^−^ and Nu-FAM83H^−^/Nu-PANX2^+^ subgroups (Table 5).

**Table 5:** Univariate and multivariate analysis of the overall survival and relapse-free survival according to the co-expression patterns of nuclear FAM83H and nuclear PANX2 expression.

**Characteristics**	**No**.	**OS**		**RFS**	
		**HR (95% CI)**	***P***	**HR (95% CI)**	***P***
**UNIVARIATE ANALYSIS**					
Nu-FAM83H/Nu-PANX2, −/− or −/+	142/199	1	<0.001	1	<0.001
+/−	31/199	2.840 (0.928−8.686)	0.067	2.922 (1.210−7.053)	0.017
+/+	26/199	10.779 (4.513−25.745)	<0.001	7.951 (3.754−16.844)	<0.001
**MULTIVARIATE ANALYSIS**					
Age, > 55 (vs. ≤ 55)		3.058 (1.027−9.104)	0.045		
TNM stage, II-IV (*vs*. I)		2.223 (0.967−5.114)	0.06	4.545 (2.272−9.089)	<0.001
Necrosis, presence (vs. absence)		2.417 (1.013−5.768)	0.047		
Nu-FAM83H/Nu-PANX2, −/− or −/+		1	<0.001	1	<0.001
+/−		2.799 (0.911−8.607)	0.072	2.595 (1.072−6.280)	0.034
+/+		7.242 (2.876−18.237)	<0.001	4.903 (2.230−10.780)	<0.001

*The variables included in the multivariate analysis was sex, age, tumor size, tumor stage, LN metastasis, histologic nuclear grade, tumor necrosis, and co-expression patterns of Nu-FAM83H and Nu-PANX2*.

### FAM83H Expression Is Associated With PANX2 Expression and Proliferation of Renal Cell Carcinoma Cells

**Figure 4:**
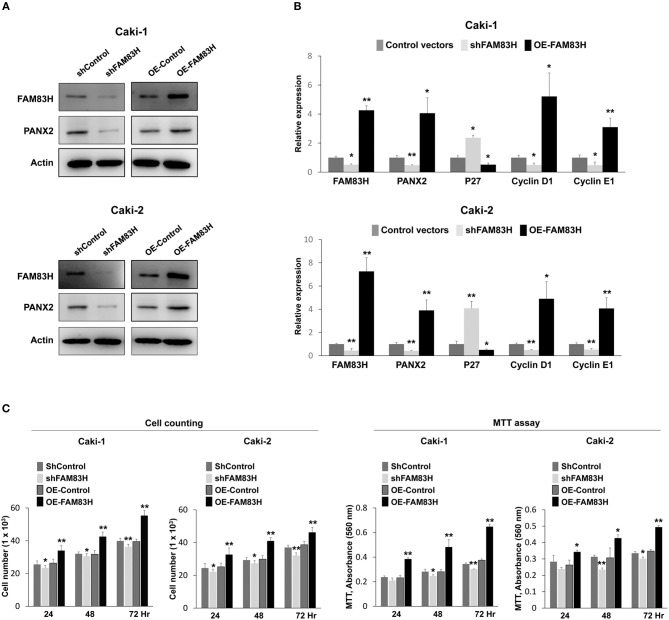
The effect of SIRT6 on the proliferation of CCRCC cells. **(A)** Western blotting for FAM83H, PANX2, and actin after knock-down of FAM83H or overexpression of FAM83H in Caki-1 and Caki-2 CCRCC cells. **(B)** Quantitative reverse-transcription polymerase chain reaction for FAM83H, PANX2, P27, cyclin D1, and cyclin E1 after knock-down of FAM83H or overexpression of FAM83H in Caki-1 and Caki-2 CCRCC cells. **(C)** Counting the number of cells and an MTT assay were performed to evaluate the effects of knock-down or overexpression of FAM893H on proliferation of Caki-1 and Caki-2 CCRCC cells. The *P* values were calculated by student's *t*-test. OE; overexpression, *vs. control, *P* < 0.05, **vs. control, *P* < 0.001.

In human CCRCC tissue, there was a significant association between FAM83H expression and PANX2 expression. Therefore, we evaluated PANX2 expression after inducing knock-down or overexpression of FAM83H in human CCRCC cells. In Caki-1 and Caki-2 CCRCC cells, knock-down of FAM83H with shRNA for FAM83H decreased expression of protein and mRNA of PANX2 and overexpression of FAM83H increased expression of protein and mRNA of PANX2 (Figures 4A,B). In addition, the expression of FAM83H was associated with the expressions of proliferation-related signaling molecules. Increased expression of FAM83H mRNA was associated with increased expression of mRNA of cyclin D1 and cyclin E1, and decreased expression of P27 mRNA (Figure 4B). Moreover, knock-down of FAM83H inhibited proliferation of CCRCC cells, and overexpression of FAM83H increased proliferation of RCC cells (Figure 4C).

## Discussion

This study investigated the expression and clinical significance of FAM83H and PANX2 expressions in CCRCC and showed that the expression patterns of FAM83H and PANX2 were significantly associated with advanced clinicopathologic factors of CCRCC such as tumor size and tumor stage. In addition, there was a significant association between FAM83H expression and PANX2 expression and the expression patterns of both were significantly associated with shorter OS and RFS of CCRCC patients. Especially, the nuclear expression of FAM83H was an independent indicator of shorter OS and RFS of CCRCC patients in the multivariate analysis. Moreover, the co-expression patterns of Nu-FAM83H and Nu-PANX2 were significantly associated with OS and RFS of CCRCC patients. In addition, the expression of FAM83H was associated with PANX2 expression and proliferation of CCRCC cells. Therefore, this study suggests that FAM83H and PANX2 might be involved cooperatively in the progression of CCRCCs and their expression patterns are useful for the prediction of survival of CCRCC patients.

The most significant result of this study is that the nuclear expression of FAM83H was an independent indicator of shorter OS and RFS of CCRCC patients in the multivariate analysis. Similarly, both nuclear and cytoplasmic expression of FAM83H (as indicated by immunohistochemical staining) predicted shorter survival of hepatocellular carcinoma patients (6). In a recent study on FAM83 family genes in various human cancers, the FAM83H gene was consistently overexpressed in lung, breast, colorectal, liver, ovary, pancreas, and gastric cancers, and higher expression of the FAM83H gene was associated with shorter survival of uterine cancer (1). However, FAM83H gene expression was decreased in astrocytoma and oligodendroglioma of the brain, and downregulation of FAM83H was associated with favorable prognosis of glioblastoma and head and neck cancer (1). The data for FAM83H expression in CCRCC is limited. However, when we searched OncoLnc public database (http://www.oncolnc.org), higher expression of FAM83H RNA was associated with shorter OS of CCRCC (Log-rank, *P* = 0.001) (Supplementary Figure 1). Therefore, although there are limited reports for the prognostic role of FAM83H expression, our results suggest that FAM83H expression might be used as a prognostic marker for CCRCC patients.

Concerning the mechanism how FAM83H is involved in tumorigenesis, the role of FAM83H in the proliferation of cancer cells has been suggested in prostatic cancer cells (8). We observed that knock-down of FAM83H inhibited proliferation of CCRCC cells and overexpression increased proliferation of CCRCC cells. FAM84H mediated proliferation of CCRCC cells was associated with the expression of cyclin D1, cyclin E1, and P27. In line with our results, in a previous study knock-down of FAM83H inhibited the colony forming ability of LNCaP prostate cancer cells (8). In colorectal cancer cells, knock-down of FAM83H inhibited migration by disrupting keratin cytoskeleton organization (9). In addition, FAM83H is expected to have an oncogenic role because its transcription is regulated by the oncogene MYC and it mediates MYC-related proliferation of hepatocellular carcinoma cells (6, 20–22). Knock-down of FAM83H inhibited the proliferation and invasiveness of hepatocellular carcinoma cells (6). FAM83H overexpression in hepatocellular carcinoma cells mediated the proliferation and invasiveness of the cells by increasing the expression of cyclin D1, cyclin E1, snail, and MMP2 (6). The fact that FAM83H-mediated increased the expression of snail and MMP2, and increased the invasiveness of hepatocellular carcinoma cells suggests that FAM83H might be involved in the epithelial-to-mesenchymal transition of cancer cells (6). The involvement of FAM83H in the epithelial-to-mesenchymal transition has also been suggested in colorectal cancer because overexpression of FAM83H induced nuclear localization of β-catenin and suppressed E-cadherin expression (9). Therefore, the significant association between Nu-FAM83H expression and shorter survival of CCRCC might be related with the oncogenic roles of FAM83H in the proliferation and invasiveness of cancer cells (23–25). However, further study is needed to clarify the role of FAM83H in human cancers.

In this study, FAM83H was observed to be expressed in both the cytoplasm and nuclei of CCRCC cells. Furthermore, in multivariate analysis, the nuclear expression of FAM83H was more predictive for the estimation of survival of CCRCC patients compared with the cytoplasmic expression of FAM83H. However, the nuclear expression of FAM83H was not the predicted expression pattern of FAM83H (7, 9, 26); a previous report indicated an exclusively cytoplasmic localization of FAM83H in HEK293 and HeLa cells (26). The public database of The Human Protein Atlas (THPA, https://www.proteinatlas.org) also reported FAM83H expression to be in the cytoplasmic membrane and cytoplasm. In colorectal cancer cells, FAM83H expression was localized in the cytoplasm in association with keratin cytoskeleton structures (9). However, a subsequent report showed that FAM83H localizes to the nuclei when it is overexpressed, and nuclear localization of FAM83H required SON (27). Moreover, nuclear localization of FAM83H was associated with disruption of the keratin cytoskeleton, and this finding suggested that FAM83H might be involved in the epithelial-to-mesenchymal transition of colorectal cancers (27). In addition, FAM83H could translocate to nuclei from the cytoplasm when mutated (28, 29). Moreover, mutant FAM83H expression in the nuclei increased nuclear localization of β-catenin in LS8 ameloblast cells (30). Therefore, nuclear localization of mutant FAM83H plays a role in the inhibition of mineralization of LS8 ameloblast cells *via* nuclear localization of β-catenin (30). However, when considering the role of β-catenin in tumor progression, especially when it is localized in nuclei (31–34), mutant FAM83H-mediated nuclear localization of β-catenin supports the hypothesis that mutant FAM83H is involved in tumorigenesis. However, mutation of FAM83H itself could not explain all for the nuclear localization of FAM83H. When we searched the cBioPortal database (http://www.cbioportal.org, accession date; 26 August 2018) (11, 12), the mutation of FAM83H in CCRCC was very low and ranged from 0.19% (1 of 538 cases) to 0.20% (1 of 499 cases). In our results, Nu-FAM83H positivity was seen in 29% (57/142) of CCRCCs. Therefore, in addition to the mutational status of FAM83H, these findings suggest that higher expression of FAM83H itself and/or the microenvironment inducing nuclear localization of FAM83H might be involved in the progression of cancer cells. Consistently, positivity of Nu-FAM83H expression was seen in 42% (68/163) of cases of human hepatocellular carcinoma and Nu-FAM83H expression was significantly associated with the presence of vascular invasion and higher tumor stage (6). Therefore, these results suggest that nuclear localization might have a role in tumorigenesis.

Nuclear and/or cytoplasmic expression of PANX2 was significantly associated with shorter OS and RFS of CCRCC patients in the univariate analysis, as well as with various poor clinical-pathologic prognostic parameters such as older age, larger tumor size, higher tumor stage, lymph node metastasis, higher histologic grade, and tumor necrosis (Table 2). These findings suggest that PANX2 might be involved in the progression of CCRCC. However, despite the prognostic significance of Nu-PANX2 and Cy-PANX2 expression in the univariate analysis, the expression patterns of Nu-PANX2 and Cy-PANX2 were not independent indicators of survival of CCRCC patients in multivariate analysis. This might be related with the significant association between PANX2 expression and FAM83H expression in CCRCC. Therefore, when we performed multivariate analysis without including Nu-FAM83H and Cy-FAM83H expressions, Nu-PANX2 positivity was an independent indicator of shorter OS (*P* = 0.004) and RFS (*P* = 0.015). The patients with Nu-PANX2-positive CCRCC had a 3.186-fold (95% CI; 1.450–7.002) greater risk of cancer-related death and a 2.302-fold (95% CI; 1.180–4.492) greater risk of relapse or cancer-related death compared with the patients with Nu-PANX2-negative CCRCC. Therefore, despite limited reports on the role of PANX2 in human cancers, our results suggest PANX2 as a potential biologic marker of CCRCC. In addition, when we searched the OncoLnc database (http://www.oncolnc.org), higher expression of PANX2 RNA was associated with shorter OS of CCRCC patients (Supplementary Figure 1). CCRCC patients with high PANX2 RNA levels had a 2.571-fold (95% CI; 1.902–3.475, *P* < 0.001) greater risk of death compared to CCRCC patients with low expression of PANX2 RNA. However, in contrast, PANX2 inhibited proliferation and tumor formation of C6 glioma cells in *in vitro* and *in vivo* (35). Therefore, further study is needed to clarify the role of PANX2 in human cancers.

In addition to the prognostic significance of PANX2 expression in CCRCCs, our results showed that PANX2 is expressed in both the cytoplasm and nuclei of CCRCC cells. However, the nuclear expression of PANX2 was also unexpected because the expression of PANX2 has been reported to be restricted to the cytoplasm of specific tissue types (13, 36). However, in our study, 24% (48/199) and 27% (53/199) of cases of CCRCCs were classified as Nu-PANX2-positive and Cy-PANX2-positive groups, respectively. In addition, Nu-PANX2- and Cy-PANX2-positivity was significantly associated with advanced clinicopathological factors and shorter survival of CCRCCs in the univariate analysis. These results suggest that the expression pattern of PANX2 might vary according to the progression of human cancers. However, the role of nuclear PANX2 is not clear at present. One possibility is that the localization of PANX2 might be related to its post-translational stabilization and transportation to nuclei. If there is elevated expression of PANX2, it may escape degradation in the cytoplasm, and translocate to nuclei; potential support for this hypothesis may be derived from reports that the transcriptional activity of PANX2 was not correlated with protein levels of PANX2 between various tissue types of mice (14). To clarify the role of PANX2 in tumorigenesis according to its subcellular localization, further study is needed.

Another interesting finding of our study is that the co-expression patterns of FAM83H and PANX2 were very useful for the estimation of survival of CCRCC patients. Especially, the Nu-FAM83H^+^/Nu-PANX2^+^ group showed the shortest survival time. Although there has been no study which specifically investigated the relationship between FAM83H and PANX2, these findings suggest that there is a molecular relationship between FAM83H and PANX2 in the progression CCRCC. This might be related with the significant association between PANX2 expression and FAM83H expression in CCRCC as shown in Table 2 and the cBioPortal database (http://www.cbioportal.org, Pearson's correlation; 0.71, Spearman's correlation; 0.36) (11, 12). Moreover, in Caki-1 and Caki-2 CCRCC cells, knock-down of FAM83H decreased mRNA and protein levels of PANX2 and overexpression of FAM83H increased mRNA and protein levels of PANX2 (Figure 4). In addition, when we compared FAM83H RNA and PANX2 RNA expression with the data from the OncoLnc database, there was a significant positive correlation (Pearson correlation coefficient = 0.665) (Supplementary Figure 2). Therefore, further study of the molecular relationship of FAM83H and PANX2 might be helpful in understanding the progression of CCRCCs.

In conclusion, this study presents the roles for FAM83H and PANX2 in CCRCCs and suggests that FAM83H and PANX2 are closely associated and involved in the progression of CCRCCs. Especially, the expression patterns of FAM83H and PANX2 were significantly associated with shorter survival of CCRCC patients. In addition, the co-expression patterns of FAM83H and PANX2 were also significantly associated with the survival of CCRCC patients. Therefore, individual and co-expression patterns of FAM83H and PANX2 might be useful prognostic indicators for CCRCC patients. Furthermore, understanding for the role of FAM83H in conjunction with PANX2 might be helpful in establishing a new therapeutic strategy for CCRCCs.

## Author Contributions

KMK, UH, JB, S-HP, KSK, SH, HP, HL, MC, WM, MK, and KJ participated in the study design. KMK, UH, and JB performed the experiment. KMK, UH, JB, S-HP, KSK, SH, HP, HL, MC, WM, MK, and KJ were involved in data collection and data interpretation. KMK, UH, JB, S-HP, KSK, SH, HP, MC, WM, and KJ participated in the statistical analyses. KMK, UH, JB, S-HP, KSK, SH, HP, HL, MC, WM, MK, and KJ wrote the manuscript. All authors read and approved the final manuscript.

### Conflict of Interest Statement

The authors declare that the research was conducted in the absence of any commercial or financial relationships that could be construed as a potential conflict of interest.
