# Supplementary material for: The Expression Patterns of FAM83H and PANX2 Are Associated With Shorter Survival of Clear Cell Renal Cell Carcinoma Patients
Source: Front Oncol. 2019 Jan 22;9:14. doi: 10.3389/fonc.2019.00014 (PMC6349742; doi:10.3389/fonc.2019.00014)
Supplement: Supplementary file 1 [file Table_1.DOCX]

Supplementary Material

The expression patterns of FAM83H and PANX2 are associated with shorter survival of clear cell renal cell carcinoma patients

**Kyoung Min Kim****^1^****^, 2, 3, 4†^, Usama Khamis Hussein^1, 5†^, Jun Sang Bae^1, 2, 3†^, See-Hyoung Park^6^, Keun Sang Kwon^7^, Sang Hoon Ha^8^, Ho Sung Park^1, 2 , 3^, Ho Lee^9^, Myoung Ja Chung^1, 2, 3^ Woo Sung Moon^1, 2, 3^, Myoung Jae Kang^1, 2, 3^, Kyu Yun Jang^1, 2 , 3, 4*^**

**^†^ These authors contributed equally to this work**

***Correspondence:**

Kyu Yun Jang

kyjang@chonbuk.ac.kr,

**SUPPLEMENTARY FIGURE LEGEND**

**SUPPLEMENTARY FIGURE S1** Survival analysis according to the RNA expression of FAM83H and PANX2. Kaplan-Meier survival analysis according to the RNA expression of FAM83H (**A**) and PANX2 (**B**) in clear cell renal cell carcinoma. The data for RNA levels of FAM83H and PANX2, and survival data of clear cell renal cell carcinoma patients were obtained from the OncoLnc database (http://www.oncolnc.org. Accessed 26 August 2018).

**SUPPLEMENTARY FIGURE S2** The association between RNA expression of FAM83H and PANX2 in clear cell renal cell carcinomas. The data for RNA levels of FAM83H and PANX2, and survival data of clear cell renal cell carcinoma patients were obtained from the OncoLnc database (http://www.oncolnc.org. Accessed 26 August 2018).
