# Supplementary figures and images for: The Expression Patterns of FAM83H and PANX2 Are Associated With Shorter Survival of Clear Cell Renal Cell Carcinoma Patients
Source: Front Oncol. 2019 Jan 22;9:14. doi: 10.3389/fonc.2019.00014 (PMC6349742; doi:10.3389/fonc.2019.00014)

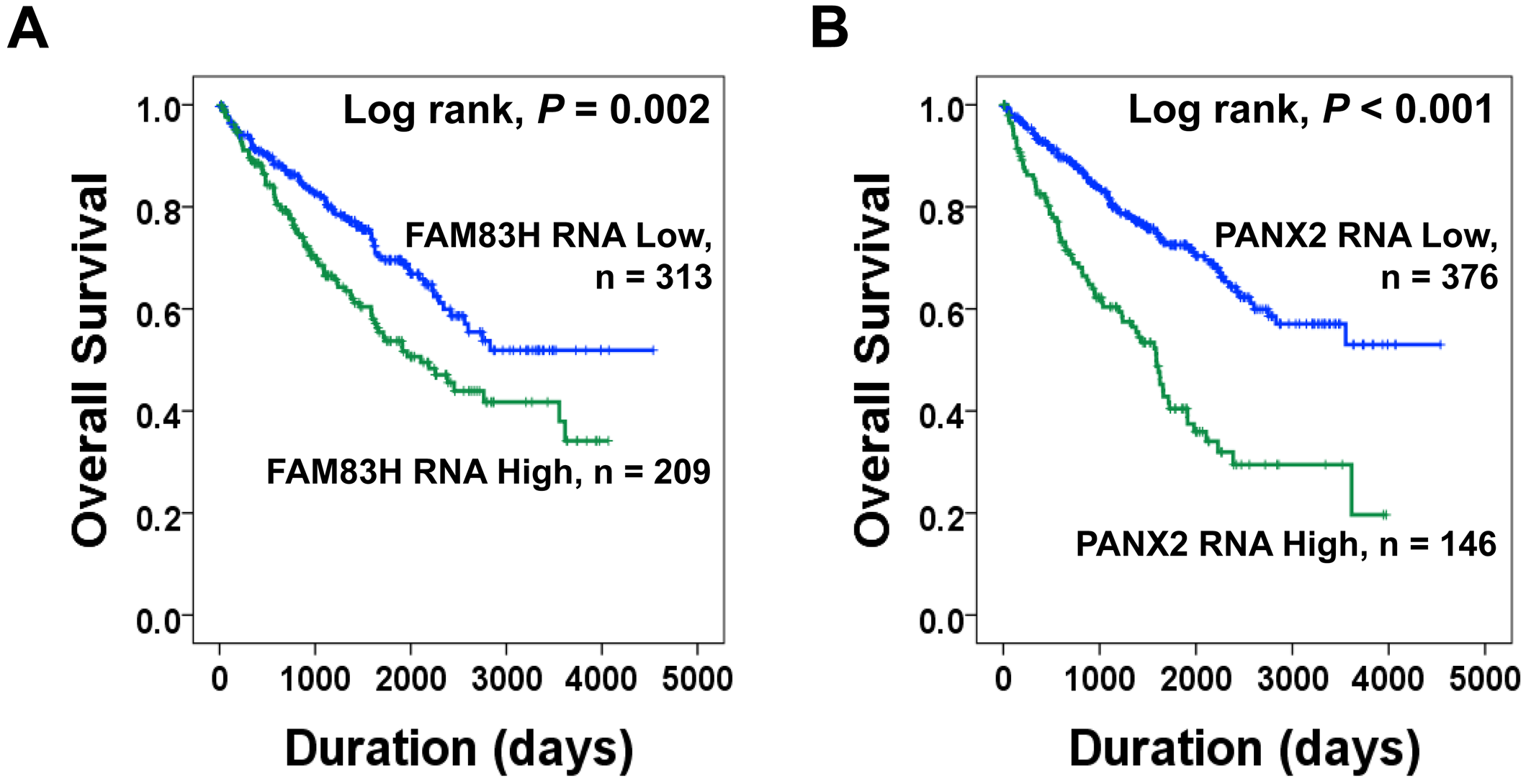

Supplement: Supplementary file 2 [file Image_1.TIF]

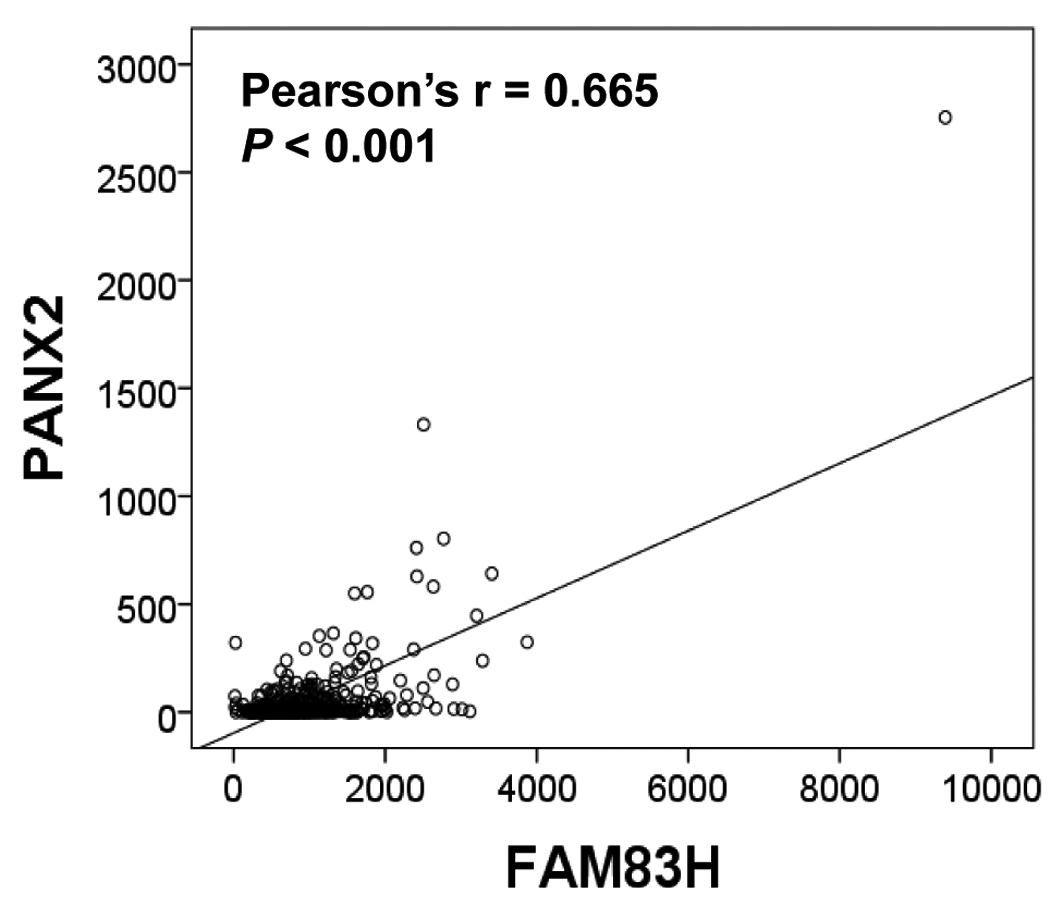

Supplement: Supplementary file 3 [file Image_2.TIF]
